# The importance of dystrophin and the dystrophin associated proteins in vascular smooth muscle

**DOI:** 10.3389/fphys.2022.1059021

**Published:** 2022-11-25

**Authors:** Katherine M. Kaplan, Kathleen G. Morgan

**Affiliations:** Department of Health Sciences Molecular Biology, Cell Biology & Biochemistry Program, Boston University, Boston, MA, United States

**Keywords:** dystrophin, dystrophin (DMD), dystrophin (dystroglycan) expression, dystroglycan (DG), vascular, smooth muscle

## Abstract

This review details the role of dystrophin and the dystrophin associated proteins (DAPs) in the vascular smooth muscle. Dystrophin is most comprehensively studied in the skeletal muscle due to serious symptoms found related to the skeletal muscle of patients with muscular dystrophy. Mutations in the dystrophin gene, or DAPs genes, result in a wide range of muscular dystrophies. In skeletal muscle, dystrophin is known to act to as a cytoskeletal stabilization protein and protects cells against contraction-induced damage. In skeletal muscle, dystrophin stabilizes the plasma membrane by transmitting forces generated by sarcomeric contraction to the extracellular matrix (ECM). Dystrophin is a scaffold that binds the dystroglycan complex (DGC) and has many associated proteins (DAPs). These DAPs include sarcoglycans, syntrophins, dystroglycans, dystrobrevin, neuronal nitric oxide synthase, and caveolins. The DAPs provide biomechanical support to the skeletal or cardiac plasma membrane during contraction, and loss of one or several of these DAPs leads to plasma membrane fragility. Dystrophin is expressed near the plasma membrane of all muscles, including cardiac and vascular smooth muscle, and some neurons. Dystrophic mice have noted biomechanical irregularities in the carotid arteries and spontaneous motor activity in portal vein altered when compared to wild type mice. Additionally, some studies suggest the vasculature of patients and animal models with muscular dystrophy is abnormal. Although the function of dystrophin and the DAPs in vascular smooth muscle is not thoroughly established in the field, this review makes the point that these proteins are expressed, and important and further study is warranted.

## Introduction

### Dystrophin

Dystrophin is the largest known human gene and is located on the X chromosome (Tennyson et al., 1995). The gene contains 79 exons, codes for a 14 kb mRNA and a 427 kDa protein (Hoffman et al., 1987; Koenig et al., 1988). The dystrophin protein is comprised of four domains: 1) the actin-binding domain (ABD) at the amino terminal domain 2) the central rod domain that contains 24 triple helical spectrin-like repeats (SR) combined with four hinge domains 3) the cysteine-rich domain 4) the carboxy-terminal domain (Koenig et al., 1988). The dystrophin protein is known to be associated with the dystroglycan complex (DGC) and thus links the extracellular matrix (ECM) to the underlying actin cytoskeleton (Campbell and Kahl, 1989). Dystrophin is also known to act to as a cytoskeletal stabilization protein and defends cells against damage due to contraction (Weller et al., 1990). Dystrophin stabilizes the plasma membrane by transmitting forces generated by sarcomeric contraction to the ECM (Weller et al., 1990; Moens et al., 1993; Vilquin et al., 1998). Skeletal muscles lacking functional dystrophin are mechanically vulnerable; and contraction of the cells results in membrane damage (Weller et al., 1990).

Dystrophin is expressed at the plasma membrane of all muscles, including cardiac, smooth, and skeletal muscle, as well as some neurons (Arahata et al., 1988; Hoffman et al., 1988; Byers et al., 1991; Ahn and Kunkel, 1993). Mutations in the dystrophin gene lead to either Duchenne Muscular Dystrophy (DMD) or Becker Muscular Dystrophy (Hoffman et al., 1987; Bonilla et al., 1988). Out-of-frame mutations of 1 or several of the 79 exons in the full-length dystrophin gene results in a lack of a functional protein, causing the DMD phenotype (Hoffman et al., 1987; Bonilla et al., 1988). DMD is inherited in an X-linked recessive manner and therefore is most prevalent in males however female carriers can also have symptoms due to X-inactivation (Nozoe et al., 2016). The majority of DMD mutations are inherited; however spontaneous mutations account for 30% of DMD cases (Dent et al., 2005). DMD is fatal and currently incurable (Khurana and Davies, 2003).

### Dystrophin associated proteins

**FIGURE 1:**
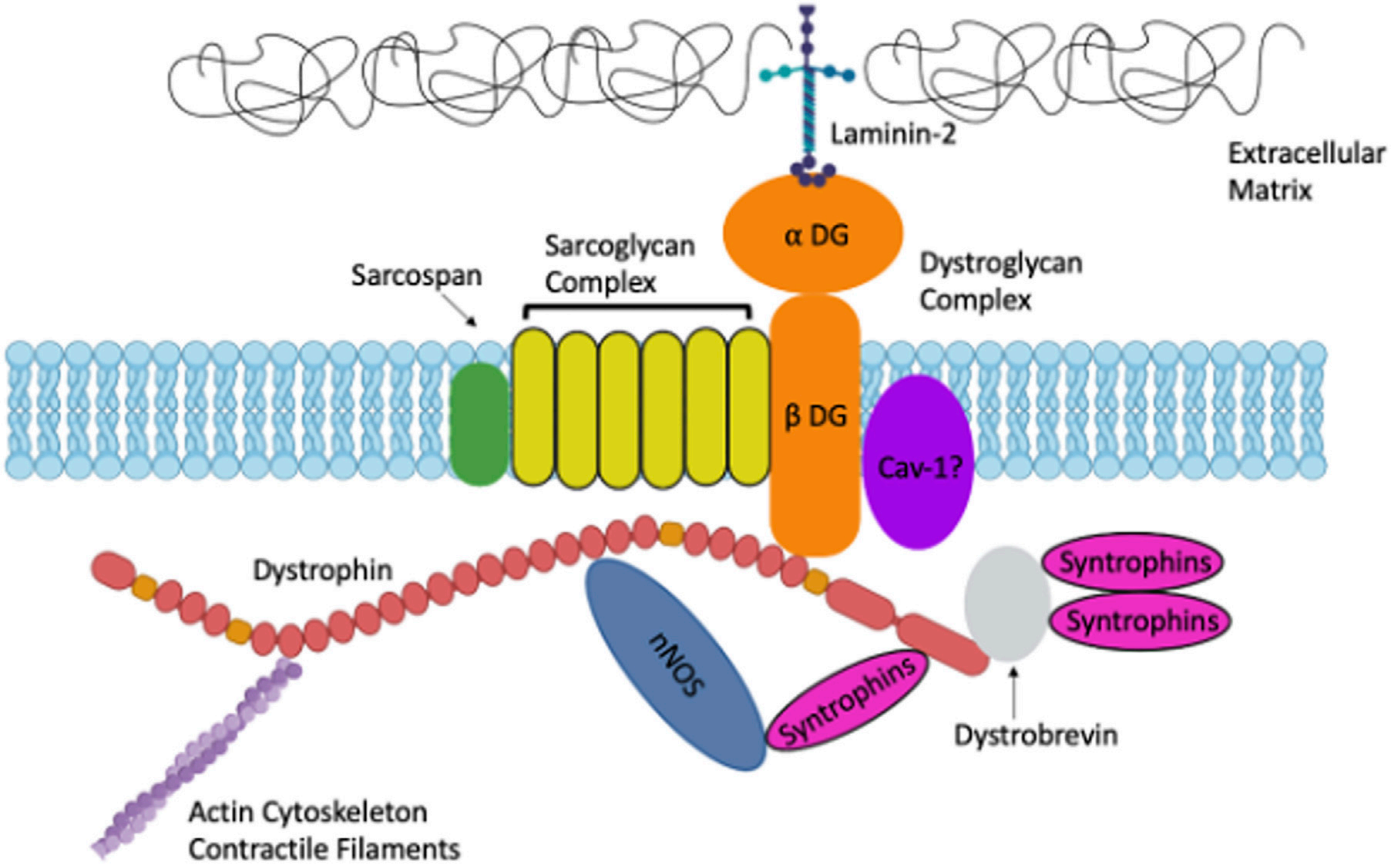
A. Schematic representation of Dystrophin and the Dystrophin-Associated Proteins in muscle including: Dystroglycans, Sarcoglycans, Syntrophins, Caveolins and more. Created with biorender.com.

Dystrophin is also an important scaffold for dystrophin associated proteins (DAPs) including sarcoglycans, syntrophins, dystroglycans, dystrobrevin, neuronal nitric oxide synthase (nNOS) and caveolins (Winder, 2001; Khurana and Davies, 2003). DAPs provide biomechanical support to the muscle plasma membrane during contraction, and loss of 1 or several of these proteins leads to plasma membrane fragility (Winder, 2001). Sarcoglycans, syntrophins, dystroglycans, dystrobrevin, and neuronal nitric oxide synthase comprise the dystroglycan complex (DGC) (Winder, 2001; Khurana and Davies, 2003) (Figure 1). Genetic mutations in DAPs can result in other types of muscular dystrophies such as Limb Girdle Muscular Dystrophy (Khurana and Davies, 2003). The widely expressed α and β dystroglycans are key proteins in the DGC, linking laminin-2 and dystrophin (Ehmsen et al., 2002). Both dystroglycans are from a single post-translationally modified polypeptide and are heavily glycosylated prior to being sorted to their extracellular and transmembrane locations (Ehmsen et al., 2002). Sarcoglycans are additional DAPs expressed in both skeletal and smooth muscle (Straub et al., 1999; Ehmsen et al., 2002). Some sarcoglycans have been shown to be expressed in smooth muscle while others have been shown to be strictly expressed in skeletal muscle (Yamamoto et al., 1994; Straub et al., 1999). There are multiple sarcoglycans; some bind dystrophin directly, others bind indirectly (Ehmsen et al., 2002). Other DAPs include dystrobrevins, syntrophins, neuronal nitric oxide synthase (nNOS) and caveolins. Dystrobrevins bind dystrophin and indirectly interact with sarcoglycans (Ehmsen et al., 2002). There is very limited literature on dystrobrevins in smooth muscle, but α-dystrobrevin-1 was observed in vascular smooth muscle cells (VSMCs) (Loh et al., 2000). There are three syntrophin isoforms in skeletal muscle. Syntrophins are hypothesized to act as modular adaptors recruiting signaling proteins to the sarcolemma and DGC (Eisenberg et al., 2007). Another DAP is nNOS which interacts with syntrophin, and it is lost in several muscular dystrophies including DMD (Ehmsen et al., 2002). nNOS is an enzyme that produces nitric oxide which is important for increasing local blood flow to match the increased metabolic load of contracting muscles (Ehmsen et al., 2002). nNOS interacts directly with spectrin-like repeats 16 and 17 in the central rod domain of dystrophin (Lai et al., 2009; Tidball and Wehling-Henricks, 2014; McGreevy et al., 2015). Caveolins are also essential proteins involved in caveolae vesicular invaginations of the plasma membrane and are found in most cell types (Cohen et al., 2004). Cav-3 is muscle specific however Cav-1 is required for caveolae formation in smooth muscle (Cohen et al., 2004). Caveolins have been hypothesized to interact with dystroglycan and mutations in Cav-3 are associated with types of muscular dystrophy (Galbiati et al., 2001). Sharma et al. (2010) demonstrated that Cav-1 interacts with β-dystroglycan in airway smooth muscle.

Pubmed searches indicate that only approximately four percent of published papers on dystrophin are studies in smooth muscle. The lack of published papers addressing dystrophin in smooth muscle demonstrates the need for research in this area. Dystrophin is expressed near the plasma membrane of all muscles, including cardiac and vascular smooth muscle, as well as some neurons (Arahata et al., 1988; Hoffman et al., 1988; Byers et al., 1991; Ahn and Kunkel, 1993). Dystrophin is most comprehensively studied in the skeletal muscle due to serious symptoms found related to the skeletal muscle of patients with muscular dystrophy. Dystrophin is an important scaffold for dystrophin associated proteins (DAPs) in skeletal muscle (Winder, 2001; Khurana and Davies, 2003). In skeletal muscle, the DAPs provide biomechanical support to the muscle plasma membrane during contraction, and loss of one or several of these proteins leads to plasma membrane fragility (Winder, 2001). DAPs have been studied extensively in skeletal muscle and different types of muscular dystrophies, but more research needs to be conducted on DAPs in smooth muscle. Due to the lack of studies on dystrophin and DAPs in vascular smooth muscle, and some contradictions in the field, it is currently unknown if dystrophin and DAPs have the same role in vascular smooth muscle as that they do in skeletal muscle. It may be hypothesized that these proteins may have different functions in the vascular smooth muscle because smooth muscle and skeletal muscle are inherently different, especially in the unique the mechanisms involved in contraction. This review examines dystrophin and the DAPs in the vascular smooth muscle.

### Vascular smooth muscle differentiation

Dystrophin has been proposed to be a marker of VSMC differentiation. Lees et al. demonstrated that dystrophin could be used as a marker of functional cultured rat VSMCs because it was only expressed in contractile VSMCs and not proliferative VSMCs (Lees et al., 1994) (Table 1). In the last few years, there has been some increase in studying dystrophin in VSMCs. Turczynska et al. (2015) treated murine VSMCs with an actin-stabilizing agent, jasplakinolide, and analyzed the cells by microarrays. This group concludes that expression of dystrophin seems to be a differentiation marker, similar to expression of alpha actin or Myh11, which is upregulated by an increased F/G actin ratio. Interestingly, these authors noted that changes in the F/G actin ratio could not be demonstrated in VSMCs from mdx mice (Turczynska et al., 2015). These findings suggest actin regulates dystrophin in VSMCs and dystrophin may be a maker of VSMC differentiation.

**TABLE 1:** Key papers highlighting connection between dystrophin and vascular smooth muscle.

Authors	Article	Journal	Important findings
Mendell et al. (1971)	Duchenne muscular dystrophy: functional ischemia reproduces its characteristic lesions	*Science 1971*	Grouped necrosis in the muscles of dystrophy patients could be due to compromised capillary blood supply and ischemia
Bradley et al. (1975)	Failure to confirm a vascular cause of muscular dystrophy	*Arch Neurol 1975*	No obvious differences in blood flow or arterial occlusion are detected in human muscular dystrophy patients
Engel and Hawley. (1977)	Focal lesions of muscle in peripheral vascular disease	*Neurol* 1977	Two patients were studied and determined to have lesions in skeletal muscle due to ischemia caused by peripheral vascular disease essentially identical to the early muscle lesions of DMD patients and carriers of the disease
Koehler. (1977)	Blood vessel structure in Duchenne muscular dystrophy. I. Light and electron microscopic observations in resting muscle	*Neurology 1977*	Skeletal muscle blood vessels from eight patients with documented Duchenne type muscular dystrophy were examined by microscopy concluding vascular abnormalities are minimal and nonspecific
Miike. (1987)	Vascular endothelial cell injury and platelet embolism in Duchenne muscular dystrophy at the preclinical stage	*Neurol Sci 1987*	Capillaries in DMD patients were almost completely obstructed, they had narrow lumens, there was replication of the basement membrane around the vessels, and degenerating and regenerating capillaries were also observed
Sugino et al. (1991)	Vascular alterations in Fukuyama type congenital muscular dystrophy	*Brain Dev 1991*	Abnormalities are evident in blood vessel structure in a unique form of muscular dystrophy (Fukuyama)
Lees et al. (1994)	Dystrophin (Xp21), a new phenotype marker of cultured rat aortic myocytes	*Exp Cell Res* 1994	Dystrophin can be used as a marker of functional cultured rat vascular smooth muscle cells (VSMCs) because it is only expressed in contractile VSMCs and not proliferative VSMCs
Lees et al. (1995)	Parallel expression level of dystrophin and contractile performances of rat aortic smooth muscle	*Exp Cell Res 1995*	There is increased expression of dystrophin in the rat aortic arch and increased contractility compared to the rat aortic diaphragm
Rivier et al. (1997)	Different utrophin and dystrophin properties related to their vascular smooth muscle distributions	*FEBS Lett 1997*	Small arteries express the long form of dystrophin, whereas small veins do not
Mancinelli et al. (1999)	Mechanical properties of smooth muscle portal vein in normal and dystrophin-deficient (mdx) mice	*Exp Physiol 1999*	The dystrophic process alters the spontaneous motor activity of the mdx mouse portal vein
Loufrani et al. (2004)	Absence of dystrophin in mice reduces NO-dependent vascular function and vascular density: total recovery after a treatment with the aminoglycoside gentamicin	*Arteriosclerosis, thrombosis, and vascular biology 2004*	Immunostaining of arterioles in gracilis muscle and whole-mount imaging of tibialis anterior muscle, show a decrease in the vasculature of *mdx* mice in comparison to wild-type
Ito et al. (2006)	Smooth muscle-specific dystrophin expression improves aberrant vasoregulation in mdx mice	*Human Molecular Genetics 2006*	SMTg/mdx mice demonstrate that restored dystrophin expression in the vascular smooth muscle partially corrects the abnormal α-adrenergic vasoconstriction in exercising skeletal muscle
Dye et al. (2007)	Altered biomechanical properties of carotid arteries in two mouse models of muscular dystrophy	*J. Appl Physiol 2007*	There are biomechanical differences of the carotid arteries of two mouse models of muscular dystrophy
Turczynska et al. (2015)	Regulation of smooth muscle dystrophin and synaptopodin 2 expression by actin polymerization and vascular injury	*Arteriosclerosis, thrombosis, and vascular biology* 2015	Actin regulates dystrophin in VSMCs. It is also shown that dystrophin is highly expressed in differentiated smooth muscle compared to synthetic smooth muscle
Buscher et al. (2019)	The long dystrophin gene product Dp427 modulates retinal function and vascular morphology in response to age and retinal ischemia	*Neurochem Int 2019*	Retinal function is reduced with age in male mdx mice
Lopez et al. (2020)	Contribution of TRPC Channels to Intracellular Ca (2 +) Dyshomeostasis in Smooth Muscle From mdx Mice	*Front Physiol* 2020	VSMCs from mdx mice have a dysregulation of intracellular calcium due to overactivation of transient receptor potential canonical channels
Kodippili et al. (2021)	Dystrophin deficiency impairs vascular structure and function in the canine model of Duchenne muscular dystrophy	*J Pathol 2021*	Dystrophin plays a crucial role of maintaining structure and function of the vascular endothelium and smooth muscle in large mammals

### Biomechanical properties of vascular smooth muscle

Dystrophin has been shown to be associated with biomechanical properties of the vascular smooth muscle. Rivier et al. demonstrated that small arteries expressed the long form of dystrophin, whereas small veins did not (Table 1) (Rivier et al., 1997). This suggests that dystrophin may have a unique role in the arteries that is not required in the veins. The authors further propose that dystrophin has a mechanical function in the arteries and may provide protection from muscle membrane degradation (Rivier et al., 1997). This hypothesis agrees with the proposed role of dystrophin in skeletal muscle. Future studies are needed to fully address the differences in expression of dystrophin in arteries and veins. Mancinelli et al. (1999) investigated the biomechanical properties of smooth muscle from the portal vein. They noted severely reduced spontaneous contraction waves in response to increased stretch, reduced contractile response to acetylcholine and normal passive stress strain relationships were noted in the mdx mouse portal vein (Mancinelli et al., 1999). It was concluded from multiple biomechanical experiments that the dystrophic process alters the spontaneous motor activity of the mdx mouse portal vein (Mancinelli et al., 1999). These finding suggest that dystrophin is important to the biomechanical properties of the vascular smooth muscle. Additionally, Dye et al. (2007) concluded there were biomechanical differences of the carotid arteries of two mouse models of muscular dystrophy, the mdx model and a sarcoglycan deficient mouse model (Table 1). This group noted that in both models, the dystrophic mice had decreased distensibilities in pressure-diameter tests, elevated axial loads and stresses in axial force-length tests, and decreased *in vivo* axial stretches compared to wild-type mice (Dye et al., 2007). Taken together Dye et al. (2007) suggests that the loss of DAPs may result in adaptive biomechanical changes so that overall wall mechanics are maintained in response to normal pressures. Further studies are needed to more fully understand these proposed vascular adaptations noted in the carotid arteries of both mdx and sarcoglycan deficient mice. These studies demonstrate that dystrophin is important to the biomechanical properties of the vascular smooth muscle but still leave questions unanswered.

### Contractile response to depolarization and calcium handling

Dystrophin has been shown to be associated with the contractile response to depolarization in the vascular smooth muscle. Lees et al. (1995) showed that there was an increase in expression of dystrophin in the aortic arch and this is associated with an increase in contractility compared to the diaphragmatic part of the rat aorta (Table 1). Lees et al. (1995) exclusively studied depolarization-induced contraction *via* response to potassium chloride, while other mechanisms of contraction, such as receptor-mediated contraction were not investigated in this study. This interesting finding may suggest that dystrophin plays a role in the contractile response to depolarization of vascular smooth muscle. Turczynska et al. (2015) also reported functional aspects of mdx vessels such as impaired KCl-induced contraction and impaired alpha1-adrenoceptor-mediated contraction. Future studies are needed to more thoroughly understand the mechanisms underlying the relationship between dystrophin and the contractile response to depolarization in vascular smooth muscle. Most recently, a group has determined that VSMCs from mdx mice have a dysregulation of intracellular calcium due to overactivation of transient receptor potential canonical channels (Lopez et al., 2020). Lopez et al. (2020) demonstrated that mdx VSMCs have not only increased calcium but also increased sodium. These authors claim that mechanical stretch was able to induce the increased cation influx in addition to pharmacological activation of TRPC channels with 1-oleoyl-2-acetyl-sn-glycerol (OAG). These data suggest that the lack of dystrophin in mdx VSMC makes these cells more susceptible to contraction induced damage which is again consistent with the findings in skeletal muscle (Lopez et al., 2020). These results are seemingly contradictory to the reduced contraction observed by other groups (Rivier et al., 1997; Mancinelli et al., 1999; Dye et al., 2007; Turczynska et al., 2015). This contradiction could be due to the fact that VSMC contractility is not solely based on calcium handling, but alternative proposed mechanisms include actin availability and cytoskeletal remodeling (Kim et al., 2008). As VSMC contractility is a complicated phenomenon, and there are contradictions in the field about the effects of dystrophin on VSMC contractility, additional studies are needed to address these interesting questions.

### Vasoconstriction and vasorelaxation

Lack of dystrophin has been studied in the context of vasoconstriction and vasorelaxation. Turczynska et al. (2015) reported reduced ability of VSMCs from mdx mice to relax with nitric oxide stimulation during isometric force measurement. Kodippili et al. (2021) studied the vascular endothelium and smooth muscle in a large animal model of muscular dystrophy (Table 1). These authors report significantly reduced maximum tension induced by vasoconstrictors phenylephrine and endothelin-1 in the canine model of muscular dystrophy (Kodippili et al., 2021). Additionally, acetylcholine-mediated endothelial dependent vasorelaxation was significantly decreased, while exogenous nitric oxide induced vasorelaxation was significantly increased (Kodippili et al., 2021). These findings suggest that dystrophin may play a crucial role in maintaining structure and function of the vascular endothelium and smooth muscle. Some of these findings contradict the results of Turczynska et al. (2015), specifically the response to nitric oxide. These contradictions might be due to endothelium playing a role and the different models used, mdx mice compared to the canine model. Due to the contradictions in the field, future studies are needed to fully explain the relationship between dystrophin and the structure and function of the vascular endothelium and smooth muscle.

### Contribution of vascular defects to DMD pathology

Some studies suggest that angiogenesis, the formation of new blood vessels, is important to the pathology of muscular dystrophy (Mendell et al., 1971; Engel and Hawley, 1977; Miike et al., 1987; Sugino et al., 1991; Loufrani et al., 2004). In 1977, two patients were studied and determined to have lesions in skeletal muscle due to ischemia caused by peripheral vascular disease essentially identical to the early muscle lesions of DMD patients and carriers of the disease (Engel and Hawley, 1977). This suggests grouped necrosis in the muscles of dystrophy patients could be due to compromised capillary blood supply and ischemia (Mendell et al., 1971; Engel and Hawley, 1977). The theory that dysfunctional vasculature contributes to muscular dystrophy was debated in the field and some groups determined there was no direct evidence for severe abnormalities in blood vessel morphology and blood flow (Bradley et al., 1975; Koehler, 1977). Other groups debate that there is validity to the theory that dysfunctional vasculature contributes to the muscular dystrophy phenotype (Miike et al., 1987; Sugino et al., 1991; Loufrani et al., 2004). One group studied abnormalities in blood vessel structure in muscle biopsy specimens from DMD patients (Miike et al., 1987). They concluded that the capillaries in DMD patients were almost completely obstructed, they had narrow lumens, there was replication of the basement membrane around the vessels, and degenerating and regenerating capillaries were also observed (Miike et al., 1987). The same group also demonstrated abnormalities in blood vessel structure in a unique form of muscular dystrophy (Sugino et al., 1991). More recent work focuses on the vasculature in muscles of *mdx* mice (Loufrani et al., 2004). Immunostaining of arterioles in gracilis muscle and whole-mount imaging of tibialis anterior muscle, show a decrease in the vasculature of *mdx* mice in comparison to wild-type mice (Loufrani et al., 2004). These studies highlight that the vasculature of patients and animal models with dystrophy is abnormal and suggests that dystrophin may be important to the vascular system (Mendell et al., 1971; Engel and Hawley, 1977; Miike et al., 1987; Sugino et al., 1991; Loufrani et al., 2004) (Table 1). More recently, Bucher et al. (2019) studied retinal function and vascular morphology in response to age and retinal ischemia in mdx mice. This group concluded that retinal function was reduced with age in male mdx mice (Bucher et al., 2019). These studies indicate that the vascular system, and more importantly angiogenesis, are clearly affected in the absence of dystrophin. Other symptoms of muscular dystrophy such as cognitive impairment and cardiomyopathy could be associated with vascular smooth muscle dysfunction. Mutations in dystrophin and sarcoglycans result in different muscular dystrophies and can be associated with cardiomyopathy (Towbin, 1998; Melacini et al., 1999). One group determined in a mouse model that depletion of the sarcoglycan-sarcospan complex in vascular smooth muscle perturbs vascular function, initiates cardiomyopathy, thereby worsening muscular dystrophy (Coral-Vazquez et al., 1999). This finding suggests that the lack of DAPs in the vasculature may contribute to the DMD phenotype and the DAPs may have a functional role in vascular smooth muscle. Another interesting symptom of muscular dystrophy that may relate to vascular smooth muscle disfunction is cognitive impairment. One group studied cerebral perfusion in patients with DMD (Doorenweerd et al., 2017). It was concluded that globally reduced cerebral perfusion is found in DMD (Doorenweerd et al., 2017). Another group came to the same conclusion, that cerebral perfusion was reduced, in the mdx model mice (Goodnough et al., 2014). Although these authors did not directly investigate the molecular function of dystrophin in the vasculature in the brain, these studies suggest that dystrophin may be important to vascular function in the brain. Thus, not only does muscular dystrophy result in defective skeletal muscle and impaired vascular function but additional symptoms such as cardiomyopathy and cognitive impairment might also suggest that dystrophin and the DAPs may be important to vascular smooth muscle.

### Therapeutic strategies for DMD should consider role of dystrophin in vascular smooth muscle

Additionally, studies suggest that it may be possible to therapeutically recover patients’ poor vascular function ultimately improving the muscular dystrophy phenotype. Recently, an mouse model was generated to study smooth muscle and dystrophin. Ito et al. (2006) generated transgenic mdx mice that expresses dystrophin only in smooth muscle (SMTg/mdx). These SMTg/mdx mice demonstrated that restored dystrophin expression in the vascular smooth muscle partially corrects the abnormal α-adrenergic vasoconstriction in exercising skeletal muscle (Ito et al., 2006). These findings suggest that dystrophin in vascular smooth muscle might be functionally important. This also argues that therapeutic strategies for DMD should consider the role of dystrophin in the vascular smooth muscle. This review highlights that there is a major need for more research investigating the role of dystrophin in vascular smooth muscle, which could ultimately add to therapeutics for muscular dystrophies.
